# A latent bleeding complication in ipsilateral common femoral puncture involving delayed diagnosis: a case report

**DOI:** 10.1186/s12872-024-04006-7

**Published:** 2024-07-05

**Authors:** Tetsuya Nomura, Shiori Yoshida, Michitaka Kitamura, Kenshi Ono, Keisuke Shoji, Yukinori Kato, Naotoshi Wada, Natsuya Keira, Tetsuya Tatsumi

**Affiliations:** Department of Cardiovascular Medicine, Kyoto Chubu Medical Center, 25, Yagi-Ueno, Yagi-cho, Nantan City, Japan

**Keywords:** Case report, Endovascular treatment, Ipsilateral puncture, Common femoral artery, Drug-coated balloon, Latent bleeding, Complication

## Abstract

**Background:**

The trans femoral ipsilateral approach is often adopted for endovascular treatment (EVT) for better steerability of guidewires or better device deliverability. However, contrary to the trans femoral contralateral approach, ipsilateral antegrade puncture sometimes causes peculiar bleeding complications.

**Case presentation:**

A 76-year-old female underwent EVT for chronic occlusion of the left superficial femoral artery (SFA) via the ipsilateral antegrade approach. After guidewire passage, we inflated the drug-coated balloons, but angiography showed blood flow stasis at the mid segment of the SFA. We also ensured prolonged balloon inflation, which resulted in favorable blood flow. While trying to ensure hemostasis, the blood pressure remained decreased, but neither bleeding nor superficial hematoma were observed at the puncture site. After hemostasis was achieved, we removed the surgical drape and noticed a swelling in the mid-portion of the thigh, distant from the puncture point. We then approached the left common femoral artery (CFA) contralaterally. Angiography showed continuous bleeding from a little bit distally to the sheath insertion point that was spreading through an intramuscular space. We stopped the bleeding with balloon tamponade inside the CFA. Angiography after hemostasis demonstrated blood flow stasis at the mid-segment of the SFA, similarly as that seen before. We confirmed compression of the SFA by a large hematoma using both intra- and extra- vascular ultrasound. Therefore, we deployed a self-expandable stent at the compressed SFA position. Finally, we achieved favorable blood flow on angiography.

**Conclusion:**

We encountered a case that latent bleeding unrecognized in the surgical field persisted while prolonged inflation of DCB was conducted at just proximal SFA. We could have avoided bailout stenting by noticing the bleeding incident in a timely manner. Prediction and prevention are essential for all kinds of procedural complications in EVT.

## Background

Lower extremity peripheral arterial disease has come to be known as one of the most common life-threatening diseases. In the past few decades, marked progress in procedural techniques and newer specialized devices for endovascular treatment (EVT) has enabled the safe treatment of more complex lesions, and current guidelines worldwide recommend EVT as a promising treatment option in most situations [1, 2]. However, bleeding complications during EVT, especially related to the management of the puncture site, can sometimes result in a critical situation, and optimal strategies for intraoperative prevention and management need to be developed.

## Case presentation

**Fig. 1:**
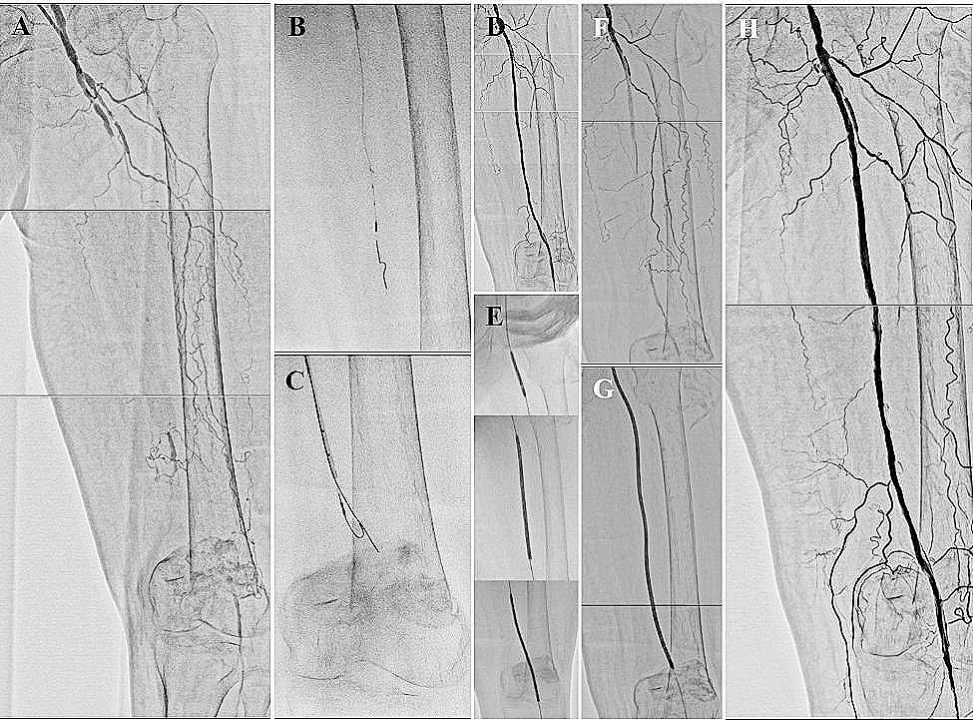
**a** Initial angiography showing a long chronic occlusion from proximal superficial femoral artery (SFA) to mid-popliteal artery. **b** Guidewire manipulation with intravascular ultrasound (IVUS) guidance. **c** Parallel wiring to track the intra-plaque area **d** Sufficient vessel dilation with favorable blood flow after prolonged balloon inflation. **e** Inflation of three drug-coated balloons (DCB) throughout the SFA. **f** Angiography post DCB inflation demonstrating blood flow stasis at the mid segment of the SFA. **g** Prolonged balloon inflation for another 5 min. **h** Final angiography showing the recovery of favorable blood flow

A 76-year-old female with a history of hypertension and dyslipidemia presented with rest-pain in her left foot (Rutherford class 4). She had previously undergone EVT of the right superficial femoral artery (SFA) because of an ischemic ulcer on the right first toe. The ankle-brachial pressure index (ABI) was 0.90 and 0.56 on the right and left ankles, respectively. Extravascular ultrasound (EVUS) of the left lower extremity showed an occlusion in the proximal segment of the SFA. She was prescribed dual antiplatelet therapy for EVT and other drugs for managing comorbidities. However, her symptoms of resting pain in the left foot were refractory to medication. Therefore, we performed EVT of the left SFA. As the starting point of the occlusion was slightly distant from the femoral bifurcation, we opted for antegrade puncture of the left common femoral artery (CFA) and inserted a 6-Fr regular 10-cm long regular sheath (Terumo Corp., Tokyo, Japan) into the left SFA. Immediately after sheath insertion, an intra-arterial bolus of 5,000 units of unfractionated heparin was administered, and the activated clotting time was controlled to ≥ 250s during the procedure. Initial angiography demonstrated longstanding chronic occlusion extending from the proximal SFA to the mid-popliteal artery (Fig. 1a). We advanced a 0.014-inch Halberd guidewire (ASAHI Intecc., Aichi, Japan) under intravascular ultrasound (IVUS) guidance using an Autobahn guiding catheter (Terumo Corp., Tokyo, Japan) (Fig. 1b). When the initial guidewire entered the subintimal space, we switched to parallel wiring to track the intra-plaque area as much as possible (Fig. 1c). After passing through the distal true lumen of the popliteal artery, prolonged inflation of the 20-cm long balloon catheter to a diameter of 5-mm resulted in sufficient vessel dilation with favorable blood flow (Fig. 1d). Therefore, we decided to finalize the procedure by inflating three drug-coated balloons (DCB) (IN.PACT Admiral; Medtronic, MN, USA) (Fig. 1e). Because the proximal lesion to be treated was distributed at the SFA ostium, we pulled back the sheath while caring for not falling out and conducted DCB inflation at the most proximal SFA for 3 min (Fig. 1e upper panel). After DCB inflation, angiography revealed blood flow stasis in the mid-segment of the SFA (Fig. 1f). Considering the possibility of an acute recoil of the SFA, we conducted prolonged balloon inflation for another 5 min (Fig. 1g), which reestablished favorable blood flow (Fig. 1h).

**Fig. 2:**
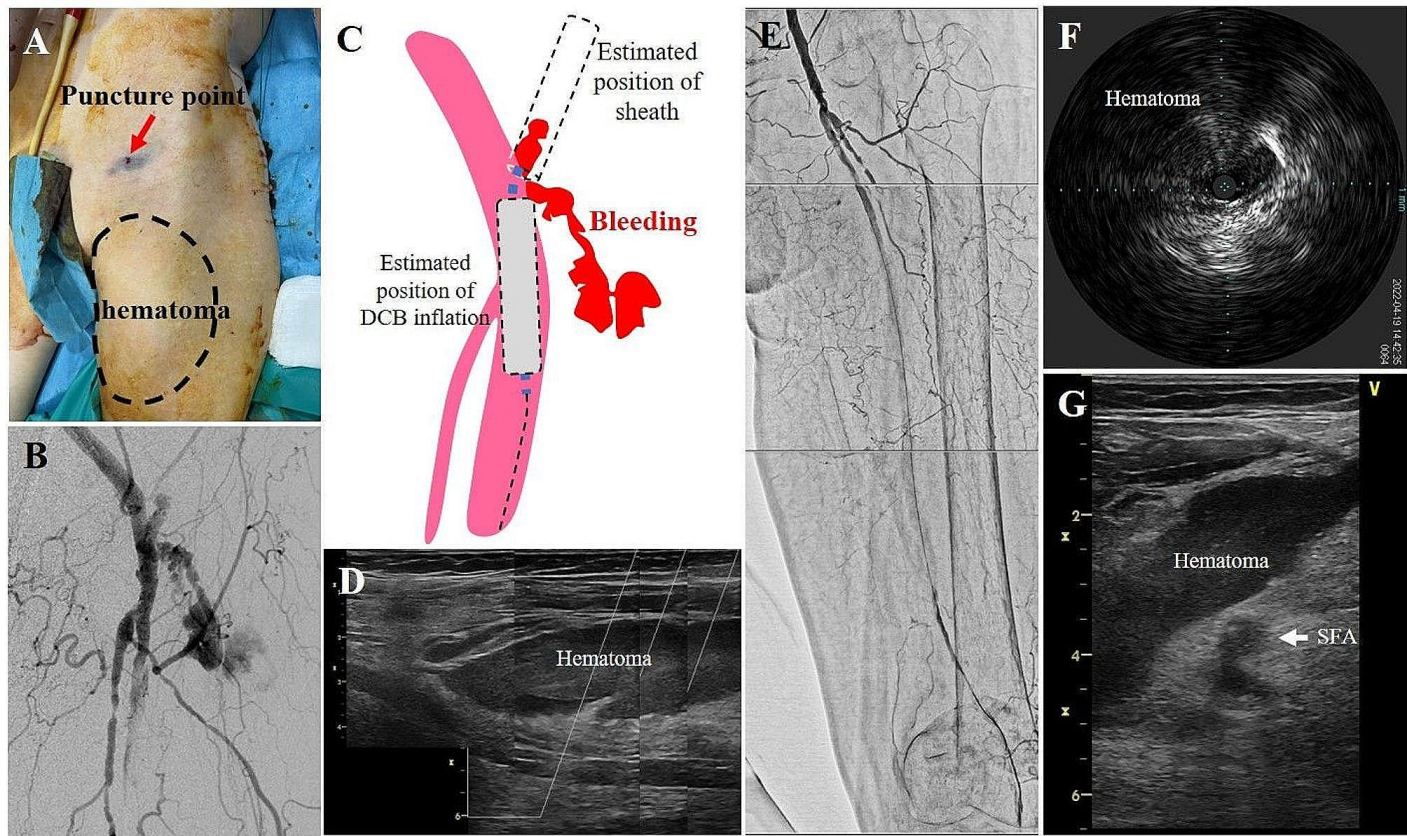
**a** Discovery of swollen thigh at the mid portion distant from the puncture point after removal of surgical drape. **b** Contralateral angiography showing continuous bleeding around the ex-punctured point and the distal spread of bleeding through an intramuscular space. **c** A schema corresponding to panel B which includes the illustration of the estimated position of the devices used in the initial EVT procedure. **d** Extravascular ultrasound (EVUS) showing a large hematoma at the swollen thigh. **e** Angiography after complete hemostasis demonstrating blood flow stasis at the mid segment of the SFA. Compressed SFA by a large hematoma observed by IVUS **f** and EVUS **g**

Hemostasis was performed using an ExoSeal (Cordis, FL, USA) followed by 5 min of manual compression. During manual compression, the patient’s systolic blood pressure decreased to approximately 70–80 mmHg. However, neither bleeding nor a superficial hematoma was observed at the puncture site. After achieving complete hemostasis, we removed the surgical drape and noticed the swollen thigh at its mid-portion, distant from the puncture point (Fig. 2a). We considered the possibility of an arterial perforation at a site other than the puncture site. We then punctured the right groin and contralaterally approached the left CFA. Angiography showed continuous bleeding from a little bit distally to the sheath insertion point that was spreading through an intramuscular space (Fig. 2b). Figure 2c shows a schema corresponding to the angiographical image of Fig. 2b which includes the illustration of the estimated position of the initial sheath. The spread of the bleeding seemed different from the estimated space of the initial sheath (Fig. 2c). Balloon tamponade inside the CFA for 15 min worked well, and the bleeding stopped completely. EVUS showed a large hematoma in the swollen thigh, in which we could see no Doppler color signal indicating continuous bleeding into the hematoma (Fig. 2d). Angiography after complete hemostasis demonstrated blood flow stasis at the mid-segment of the SFA, similarly as that seen earlier (Fig. 2e). We confirmed compression of the SFA by a large hematoma using IVUS (Fig. 2f) and EVUS (Fig. 2g).

**Fig. 3:**
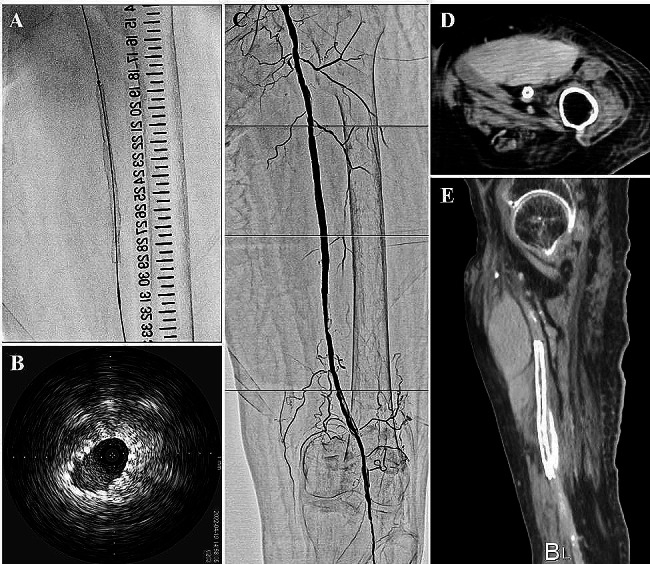
**a** Deployment of a self-expandable stent at the position of the compressed SFA **b** Cross-sectional IVUS image after stent deployment. **c** Final angiography showing favorable blood flow. Computed tomography after EVT showing a large hematoma in front of the stented SFA in the horizontal **d** and sagittal plane **e**

Therefore, we deployed a self-expandable stent at the site of compression of the SFA (Fig. 3a, b). Finally, we achieved favorable blood flow on angiography (Fig. 3c). Computed tomography after EVT revealed a large hematoma in front of the stented SFA (Fig. 3d, e). After treatment, the ABI on the left recovered to 0.90, and her symptoms of resting pain were completely resolved (Rutherford class 0). After EVT, the patient continued the same medication as before, and three months later, ABI on the left side was maintained at 0.89, and no pain in the lower extremity was observed. We observed the hematoma remaining but it was shrinking. Table 1 shows the timeline of the study patient.

**Table 1:** Timeline of the study patient

Time	Events
1 month previously	Onset of rest-pain in the left foot. ABI on the left side: 0.56.
Day 1	Endovascular treatment for left superficial femoral artery ooclusion.
	Hematoma formation in the mid-portion of the thigh.
Day 7	ABI on the left side: 0.90. Discharge from hospital with no clinical symptom.
Day 84	ABI on the left side: 0.89. Shrinked hematoma but remaining.
Day 180	Complete resolution of hematoma.

## Discussion and conclusions

We encountered a case that latent bleeding unrecognized in the surgical field persisted while prolonged inflation of DCB was conducted at just proximal SFA. Literature reveals that there have been an increasing number of cases that can be treated entirely with DCB inflation, including cases of chronic total occlusion (CTO) [3]. To complete EVT procedures with DCB inflation without requiring bailout stent deployment, the extent of arterial dissection and blood flow after lesion preparation is a critical determinant [4, 5]. For example, the occurrence of blood flow disturbance due to type E and F arterial dissection usually requires bailout stent deployment [6]. However, extravascular factors are rarely relevant to the decision of whether to deploy bailout stenting.

In our case, flow stasis in the SFA was observed immediately after DCB inflation. Although we can retrospectively imagine the cause of the static blood flow during the procedure was due to the extravascular compression by a hematoma, the swollen appearance of the thigh, which was distally distant from the puncture point, was initially concealed by the surgical drape during the procedure, and the true cause of the blood flow disturbance remained unnoticed until the end of the operation. The swelling observed in the thigh and subsequent angiography at the groin helped pinpoint the precise pathogenesis of the phenomenon. We finally overcame the blood flow disturbance caused by extravascular compression due to a large hematoma with the deployment of a self-expandable stent, but we could have avoided bailout stenting by noticing the bleeding incident in a timely manner.

When we inflated a DCB from the ostium of the SFA for 3 min, we pulled the ipsilateral sheath back as far as the common femoral puncture point, paying maximum attention to the sheath to avoid falling out the vessel. However, in retrospect, it is possible that the tip of the sheath was slightly out of the vessel, and bleeding continued during DCB inflation (Fig. 2b and c). We could see a tiny protrusion of the contrast medium but no continuous bleeding toward the estimated position of the sheath in Fig. 2b. We believe the image demonstrates the closure device well worked, and there was another origin of the continuous bleeding other than closure device failure. We suspect fistula formation, but we think it was not so severe considering the easy resolution only by balloon tamponade.

We encountered a case that latent bleeding unrecognized in the surgical field persisted while prolonged inflation of DCB was conducted at just proximal SFA. We could have avoided bailout stenting by noticing the bleeding incident in a timely manner. Bleeding complications during EVT, especially related to the management of the puncture site, can sometimes result in a critical situation. We should always keep in mind that prediction and prevention are essential for all kinds of procedural complications in EVT.

## Data Availability

The datasets used and/or analysed during the current study available from the corresponding author on reasonable request.

## Abbreviations

ABI: Ankle brachial pressure index
CFA: Common femoral artery
CTO: Chronic total occlusion
DCB: Drug-coated balloon
EVT: Endovascular treatment
EVUS: Extravascular ultrasound
IVUS: Intravascular ultrasound
SFA: Superficial femoral artery
